# Dosimetric study between a single isocenter dynamic conformal arc therapy technique and Gamma Knife radiosurgery for multiple brain metastases treatment: impact of target volume geometrical characteristics

**DOI:** 10.1186/s13014-021-01766-w

**Published:** 2021-02-27

**Authors:** Michel Chea, Karen Fezzani, Julian Jacob, Marguerite Cuttat, Mathilde Croisé, Jean-Marc Simon, Loïc Feuvret, Charles-Ambroise Valery, Philippe Maingon, Mohamed-Amine Benadjaoud, Catherine Jenny

**Affiliations:** 1 Radiation Oncology Department, Pitié-Salpêtrière Hospital, AP-HP Sorbonne University, 47-83 Boulevard de l’Hôpital, 75651 Paris Cedex 13, France; 2 Neurosurgery Department, Pitié-Salpêtrière Hospital, AP-HP Sorbonne University, Paris, France; 3 PSE-SANTE/SERAMED, Radiation Protection and Nuclear Safety Institute, Fontenay aux Roses, France

**Keywords:** Stereotactic radiosurgery, Multiple brain metastases, Single-isocenter, Gammaknife, Gradient index, Dose fall-off, Target volume effect

## Abstract

**Purpose:**

To compare linac-based mono-isocentric radiosurgery with Brainlab Elements Multiple Brain Mets (MBM) SRS and the Gamma Knife using a specific statistical method and to analyze the dosimetric impact of the target volume geometric characteristics. A dose fall-off analysis allowed to evaluate the Gradient Index relevancy for the dose spillage characterization.

**Material and methods:**

Treatments were planned on twenty patients with three to nine brain metastases with MBM 2.0 and GammaPlan 11.0. Ninety-five metastases ranging from 0.02 to 9.61 cc were included. Paddick Index (PI), Gradient Index (GI), dose fall-off, volume of healthy brain receiving more than 12 Gy (V_12Gy_) and DVH were used for the plan comparison according to target volume, major axis diameter and Sphericity Index (SI). The multivariate regression approach allowed to analyze the impact of each geometric characteristic keeping all the others unchanged. A parallel study was led to evaluate the impact of the isodose line (IDL) prescription on the MBM plan quality.

**Results:**

For mono-isocentric linac-based radiosurgery, the IDL around 70–75% was the best compromise found. For both techniques, the GI and the dose fall-off decreased with the target volume. In comparison, PI was slightly improved with MBM for targets < 1 cc or SI > 0.78. GI was improved with GP for targets < 2.5 cc. The V_12Gy_ was higher with MBM for lesions > 0.4 cc or SI < 0.84 and exceeded 10 cc for targets > 5 cc against 6.5 cc with GP. The presence of OAR close to the PTV had no impact on the dose fall off values. The dose fall-off was higher for volumes < 3.8 cc with GP which had the sharpest dose fall-off in the infero-superior direction up to 30%/mm. The mean beam-on time was 94 min with GP against 13 min with MBM.

**Conclusions:**

The dose fall-off and the V_12Gy_ were more relevant indicators than the GI for the low dose spillage assessment. Both evaluated techniques have comparable plan qualities with a slightly improved selectivity with MBM for smaller lesions but with a healthy tissues sparing slightly favorable to GP at the expense of a considerably longer irradiation time. However, a higher healthy tissue exposure must be considered for large volumes in MBM plans.

## Introduction

For the multiple brain metastases treatment, whole brain radiation therapy (WBRT) tends to disappear in favor of stereotactic radiosurgery (SRS) or stereotactic radiotherapy (SRT). One prospective study [1] indicated that there was no significant difference between two to four and five to ten metastases for the overall survival with SRS alone. No prospective studies have evaluated the use of SRS relative to whole brain radiotherapy (WBRT) for patients with more than four brain metastases. However, the current tendency is to avoid WBRT due to the attendant toxicity and neurological deterioration after such a treatment.

Historically, since 1968, Gammaknife radiosurgery is the gold standard for stereotactic treatments. The Gamma Knife (Elekta, Stockholm, Sweden) contains 192 ^60^Co sources and 4, 8 and 16 mm collimator options providing 192 narrow beams precisely focused on a target.

Other SRS techniques have been developed since using linear accelerators. Examples include multiple isocenter dynamic conformal arc therapy (MIDCA) which traditionally assigns one group of non-coplanar dynamic conformal arcs to each target, volumetric modulated arc therapy (VMAT), and CyberKnife radiosurgery (Accuray, Sunnyvale, CA, USA).

In 2016, Elements Multiple Brain Mets SRS (MBM) version 1.5 (Brainlab, Munich, Germany) revived dynamic conformal arcs as the paradigm for linac-based stereotactic radiosurgery [2–4]. Automatic treatment planning can be performed on multiple metastases simultaneously with non-coplanar dynamic conformal arcs (DCA) sharing the same isocenter (barycenter targets).

Similarly, HyperArc VMAT (Varian, Palo Alto, CA, USA) can also offer single-isocenter treatments for multiple brain metastases and seems to be a promising solution as well.

Several publications have compared the available techniques for multiple brain metastases treatments. All solutions are capable of achieving a high level of conformity; differences are found for dose spillage, planner dependency and the beam on time. Manual VMAT is reported to have a high dose spillage, a dependency on planning skills and an inter-planner variability [2, 3, 5–7]. MBM version 1.5 had a slightly lower conformity reported in few articles [5, 8] but proved a plan quality similar to MIDCA for a shorter beam delivery time [3]. Since 2019, version 2.0 is commercially available with improvements in the optimization engine and a dosimetric study compared this approach with manual VMAT [6], in which the new algorithm in MBM 2.0 compared well to VMAT, even when using multiple isocenters (one plan per target) for the latter. The authors concluded that Elements showed better plan quality in terms of selectivity and dose spillage, as well as treatment time. Another dosimetric study for MBM 2.0 compared MBM version 1.5 and 2.0 with different MLC designs [9]: HDMLC (Varian, Palo Alto, CA, USA) and Agility MLC with and without jaw tracking feature (Elekta, Stockholm, Sweden). The authors demonstrated the added values of the version 2.0 and the potential incremental benefits of improved software optimization and MLC design. HyperArc combines high conformity and high dose gradient close to the Gamma Knife with more efficiency in terms of beam-on time. [5, 8, 10, 11]. The Gamma Knife remains however a reference in terms of stereotactic treatments because of its well-known high dose gradient and high localization precision [5, 7] despite a long beam-on time and an invasive procedure to fix the mask on the skull.

All these studies were performed with a basic statistical analysis on targets of different geometrical characteristics inducing probably a bias in the comparison.

The dose spillage is often characterized by the Gradient Index but the dose fall-off is also a relevant parameter to analyze, useful in clinical conditions and considers the isodose direction. Only few articles have studied the dose fall-off; Munshi et al. [12] reported the dose fall-off pattern for the frameless SRS with 3D conformal Radiotherapy and VMAT. For both techniques, the sharpest dose fall-off was in the superior, inferior and OAR direction. It would be interesting and of a significant clinical help to have these kind of data for the other stereotactic techniques and correlate them to the widely used Gradient Index.

The authors propose a study to evaluate the improved actual version of MBM by comparison to the Gamma Knife SRS using a specific statistical method for a large number of metastases and to analyze the dosimetric impact of the target volume geometric characteristics. It also includes a dose fall-off analysis to determine the sharpest dose fall-off directions and to evaluate the Gradient Index relevancy for the dose spillage evaluation.

## Material and methods

### Patient selection

Twenty patients previously treated in our Institution between 2018 and 2019 with Gammaknife SRS (Perfexion model) were selected. The patients had three to nine brain metastases from different primary malignant tumors: lung, breast kidney or melanoma. In total, ninety-five metastases with a major axis diameter ranging from 0.3 to 4 cm and a volume ranging from 0.02 to 9.61 cc were included (Table 1). Ten of them were inside or close to the brainstem. No patient suffered from meningeal metastases.

**Table 1:** Characteristics of the lesions included in the study

Number of patients	20
Number of metastases	95
Mean volume (cc)	0.76 ± 1.35
Median volume (cc)	0.3
Volume range (cc)	0.02 to 9.61
Mean major axis diameter (cm)	1.17 ± 0.65
Major axis diameter range (cm)	0.35 to 4
Median major axis diameter (cm)	1

### Treatment planning

All patients had multimodality imaging with at least one magnetic resonance imaging (MRI) sequence dedicated for SRS (slice thickness 1.2 mm) T1 FSPGR on the 1.5 T Optima 450 MR (GE Healthcare, Chicago, IL, USA) and one computed tomography (CT) scan dedicated protocol (slice thickness 1.25 mm) on the Discovery 750 HD (GE Healthcare, Chicago, IL, USA). These sequences benefited from a quality assurance, to ensure high quality of images and minimal distortion.

All the cases were replanned using GammaPlan (GP) version 11.0 and Multiple Brain Mets version 2.0.

In order to reduce inter-operator variabiliy, GP plans were recalculated by two physicists and MBM by another single physicist.

The Gross Target Volume (GTV) was delineated using the MRI T1 FSPGR sequence. The GTV was assigned as the PTV and thus, no margin was applied. The prescribed dose was 20 Gy on the reference isodose: 50% in GP, variable from 47 to 92% automatically selected by MBM. The dose was set at 12 Gy for cases where the volume of the brainstem exposed at a dose above 12 Gy was more than 0.5 cc if 20 Gy was prescribed. The OAR constraints applied are reported in Table 2. [13–18]. Clinical constraints aimed to limit the volume of healthy brain receiving 12 Gy to 10 cc in 1 fraction.

**Table 2:** Organs at risk constraints

Optic nerves	D_max_ < 10 Gy
Eyes	D_max_ < 10 Gy
Optic chiasm	D_max_ < 8 Gy
Brainstem	V_12Gy_ < 0.5 cc
Normal brain	V_12Gy_ < 10 cc

For MBM planning on the Novalis® Truebeam™ STx (Varian, Palo Alto, CA, USA) with 6 MV photon beams (600 MU/min) and HD120 MLC, templates with number of table angles ranging from five to eight with two arc passes at each table angle were used depending on the spatial distribution of the metastases in the brain. The software automatically optimized the arc set-up geometry, MLC apertures, collimator angles and arc weighting with a couch angulation from 10° to 320° and collimator angles from 4° to 45°. Metastases lining up in the direction of leaf-motion are automatically not treated simultaneously to restrict normal tissue exposure. Each case was customized: arc lengths were selected in order to avoid eyes and to prevent isodose from surrounding two neighboring metastases, optimization volumes were set either as an organ at risk (OAR) between two lesions or as PTV to increase the coverage and two isocenters were used if metastases were far from each other.

### Plan comparison

For the plan comparison, several established dosimetric indices were used.

All the recalculated plans were accepted for a conformity index (CI) equal to 1.

The CI [19] characterizes the coverage and is defined as:

$${\rm CI}=\frac{{\text{TV}}_{{\rm PIV}}}{{\text{TV}}}$$

where ${\text{TV}}_{\rm PIV}$ is the volume of the target covered by the prescription isodose and TV is the target volume.

The selectivity is evaluated by the Paddick Index (PI) [20] which is defined as:

$${\rm PI}=\frac{{\text{TV}}^{2}_{{\rm PIV}}}{{\text{TVxPIV}}}$$

where PIV is the prescription isodose volume.

A score of 1 corresponds to an ideal isodose conformity to the target volume.

The dose spillage was evaluated by the volume of healthy brain receiving more than 12 Gy, V_12Gy,_ the dose fall-off and the Gradient Index (GI), which is defined as:

$${\rm GI}=\frac{{\text{PIV}}_{50}}{{\text{PIV}}}$$

where PIV_50_ corresponds to the volume of the half prescription isodose. The GI should be as low as possible.

**Fig. 1:**
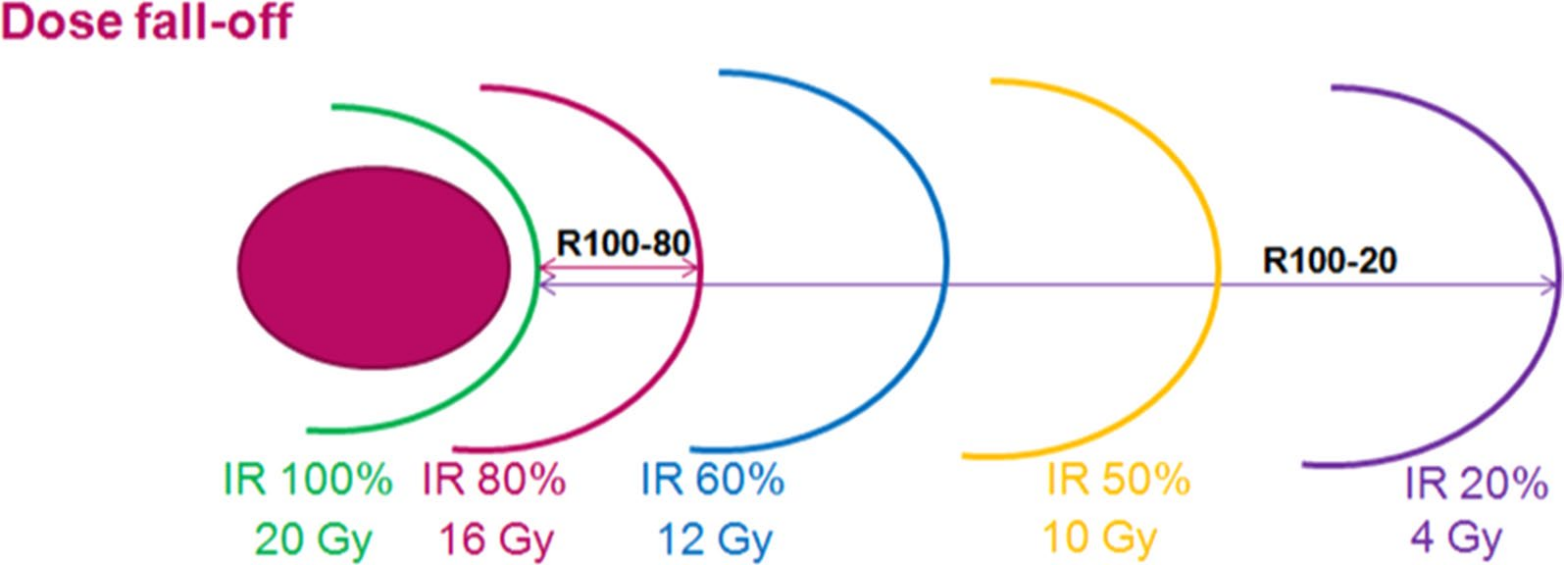
Dose fall-off (R100-80, R100-60, R100-50, R100-20) reporting in mm

The dose fall-off was expressed in %/mm. To obtain these data, the distance between the prescribed isodose (20 Gy) and the 80% (16 Gy)—60% (12 Gy)—50% (10 Gy) and 20% (4 Gy) were reported and denominated as R_100-80_, R_100-60_, R_100-50_ and R_100-20_ respectively (Fig. 1). The corresponding dose fall-off is defined as:

$${\rm Dose fall off }=\frac{\%{\;\text{of dose loss from isodose 100 to isodose X}}}{{\text{R}}_{{\rm 100-X}}}$$

where X is the percentage of the prescribed isodose.

The dose fall-off values were collected for ten lesions close to the brainstem in the anterior, posterior, left, right superior and inferior directions.

The dose volume histograms were also compared to assess the OAR sparing.

The two techniques were compared using these dosimetric indices and considered several covariates which were tumor volume, major axis diameter, Sphericity Index and distance from isocenter. The Sphericity Index (SI) is defined as the ratio of the surface of a perfect sphere having the same volume as the target volume to the surface of the target volume and reflects the complexity of the shape [21].

$${\rm SI}=\frac{{\text{A sphere}}}{{\text{A target volume}}}$$

A simple geometry getting closer to a sphere will have a Sphericity Index near 1 whereas a complex shape will decrease from 1.

This study must take into account a major clinical parameter: the duration of the treatment time. The most objective data is the beam on time since the total treatment time can variate according to the imaging verification processes associated to the therapists training. For the Gammaknife, the beam on time was rescaled to the beam on time by using new Cobalt sources reloading (3.313 Gy/min).

### Parallel study

A parallel study was led to evaluate the dosimetric impact of the isodose line (IDL) prescription available on the version 2.0 of MBM.

In the SRS Prescription mode, with controlled inhomogeneity via isodose line (IDL) prescription, the twenty selected patients were planned with the IDL prescription value set from 50 to 90% in steps of 5%.

For each plan, the mean Paddick conformity index and the mean gradient index were determined for each metastases in each plan. The mean value of the normal brain exposed to 12 Gy was determined for each plan.

### Statistical analysis

To evaluate and compare the impact of metastases covariates (tumor volume, major axis diameter, SI and distance from isocenter) on dosimetric parameters (PI, GI and the volume of the normal brain receiving at least 12 Gy) between the two techniques, a multivariate linear mixed effect regression was conducted with random effects at the metastasis level taking into account the repeated measure (a dosimetric parameter of a given metastasis is measured using the two techniques) and the patient level (clustered structure of the data since several lesions can be observed within the same patient). Due to the multivariate regression approach, the covariates effects are estimated jointly and the impact of each covariate is interpreted by keeping all the others unchanged.

The same modeling strategy was adopted to study the intra and inter technique impact of several predictors (presence of an OAR near the PTV, directions, metastasis volume and isodoses range percentages) on dose fall-off values.

The OAR dose comparisons between the two techniques were performed within a functional data analysis framework: The Dx were defined as functions on the interval [0, 1] (range of x percentages) and the inference on the difference between paired sample mean functions was conducted following Smaga et al. approach [22].

The reference here was Gamma Knife so results are expressed relative to this technique.

All analyses were performed using the MATLAB® software (version 8.2.0.701, R2013b, MathWorks©, Natick, Massachusetts).

## Results

### Parallel study

**Fig. 2:**
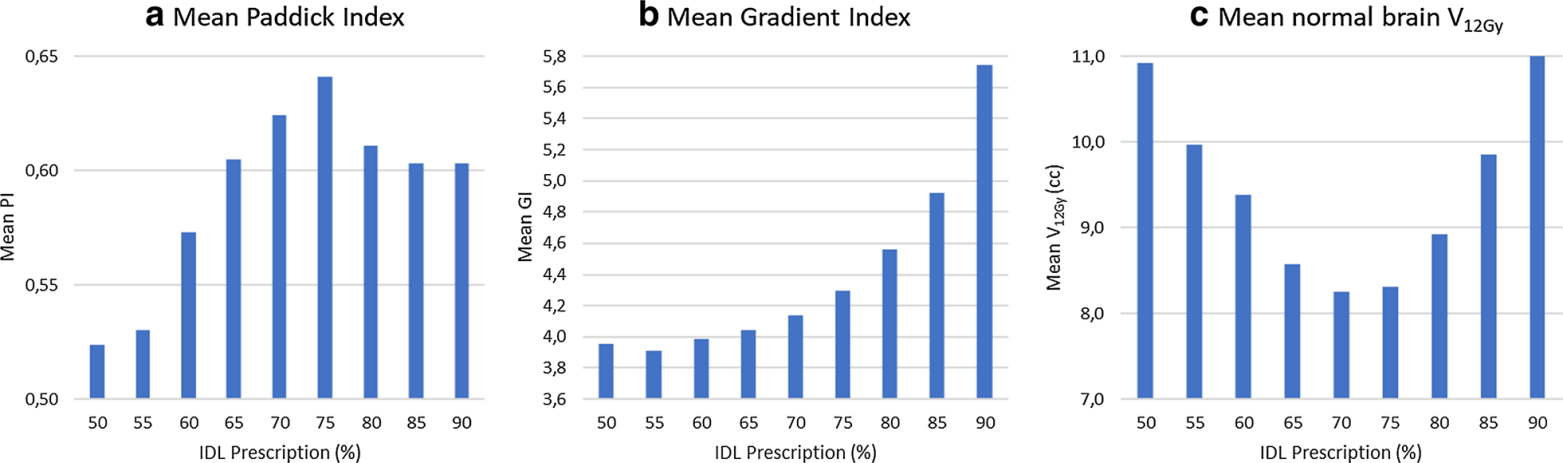
Dosimetric impact of the isodose line prescription on MBM 2.0 concerning **a** the mean Paddick Index, **b** the mean Gradient Index and **c** the mean normal brain volume receiving at least 12 Gy

In the SRS prescription mode, the average PI increased from the IDL 50% to reach a maximum at the IDL 75% and then decreased. The extreme values found were 0.52 and 0.64 for the IDL 50% and 75% respectively (Fig. 2a). The gradient index increased with the IDL with a minimum value found for IDL 55% and a maximum for IDL 90% going from 3.91 to 5.74 (Fig. 2b) whereas the volume for the normal brain receiving at least 12 Gy decreased from IDL 50% with 10.92 cc to reach a minimum value at 8.25 cc for IDL 70% and after increased until 11.72 cc for the IDL 90% (Fig. 2c). In summary, the best compromise for selectivity and sparing tissues was around a 70–75% IDL prescription.

### Plan comparison

#### Overall study

##### Dosimetric results

The mean and the near maximum (D_2%_) doses to the PTV were higher with Gamma Knife (Table 3). This difference was induced by the prescribed IDL, which is around 50% for GP and 73% for MBM. The IDL prescription automatically determined for MBM fitted well to the results of the parallel study to have the best compromise for selectivity and sparing tissues since it was between the 70 and 75% IDL prescription. The near minimum doses were equivalent since we have assured the same PTV coverage for all the plans.

**Table 3:** Doses to PTV

N = 94	MBM	GP
Mean prescription isodose (%)	73; range [47–92]	50; range [45–60]
Dnear min (Gy)	21.51 ± 1.15	21.56 ± 3.33
Dmean (Gy)	25.09 ± 2.03	30.31 ± 2.79
Dnear max	27.54 ± 2.72	38.34 ± 2.84

##### Paddick Index (PI)

For all the lesions, taking all variables together, there was no statistically significant difference for the overall mean PI (Table 4). The PI increased with the target volume. The results were in favor of MBM for the smallest target volumes but an opposite trend was observed for PTV larger than 1 cc.

**Table 4:** Dosimetric index results

	MBM	GP	*p* value
Mean PI	0.57 ± 0.12	0.532 ± 0.17	0.07
Mean GI	4.09 ± 1.14	3.22 ± 0.55	< 0.001
MeanV_12Gy_(cc)	1.87 ± 2.58	1.70 ± 2.32	0.013

A further analysis with functional data illustrated the effects of volume, major axes and sphericity on the difference between the MBM and GP in terms of PI.

**Fig. 3:**
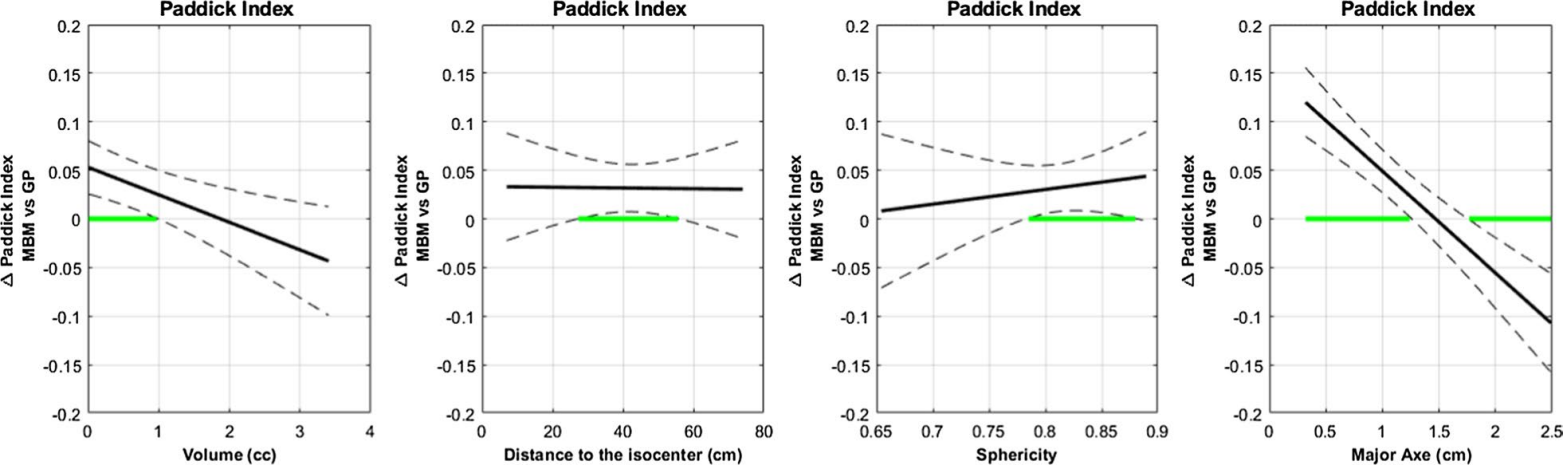
Paddick Index absolute difference between MBM and GP as a function of the volume, the distance to the isocenter, the Sphericity Index and the major axis diameter determined with a multivariate regression approach. The green segments of the abscissa axis highlight the range of values where the difference is statistically significant

In Fig. 3, the green segments of the abscissa axis highlight the range of values where the PI exhibits a significant difference between the two techniques.

With the target volume, the difference between the mean MBM PI and GP PI decreased from 0.05 to -0.05. The mean MBM PI was significantly higher for smaller target volumes (< 1 cc). The trend reversed after 1.8 cc without statistically significant differences.

With regard to the major axis diameter, the difference followed the same trend as with the volume varying from 0.1 to -0.1 and was equal to zero at 1.5 cm. The results were statistically significant and in favor of MBM for small major axes < 1.3 cm and in favor of GP for diameter > 1.75 cm.

Compared to the mean GP PI, the mean MBM PI increased with the sphericity to reach a maximum difference of 0.05 and is statistically significant for sphericity > 0.78.

For the distance to the isocenter, the mean PI difference was constantly higher of 0.04 for MBM. A statistically difference was observed between 27 and 56 mm from the isocenter.

##### Gradient Index (GI)

**Fig. 4:**
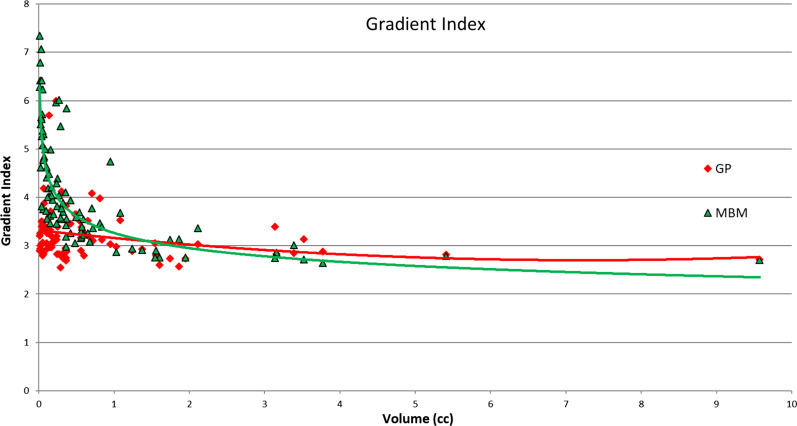
Gradient Index as a function of the volume of the PTV

The overall mean GI was 4.09 ± 1.14 for MBM and 3.22 ± 0.55 for GP (p < 0.001) (Table 4). The raw data presented in Fig. 4 demonstrates graphically that the GI decreased with increasing target volume and that the differences were mainly worse for MBM compared to GP for volumes smaller than 2 cc.

**Fig. 5:**
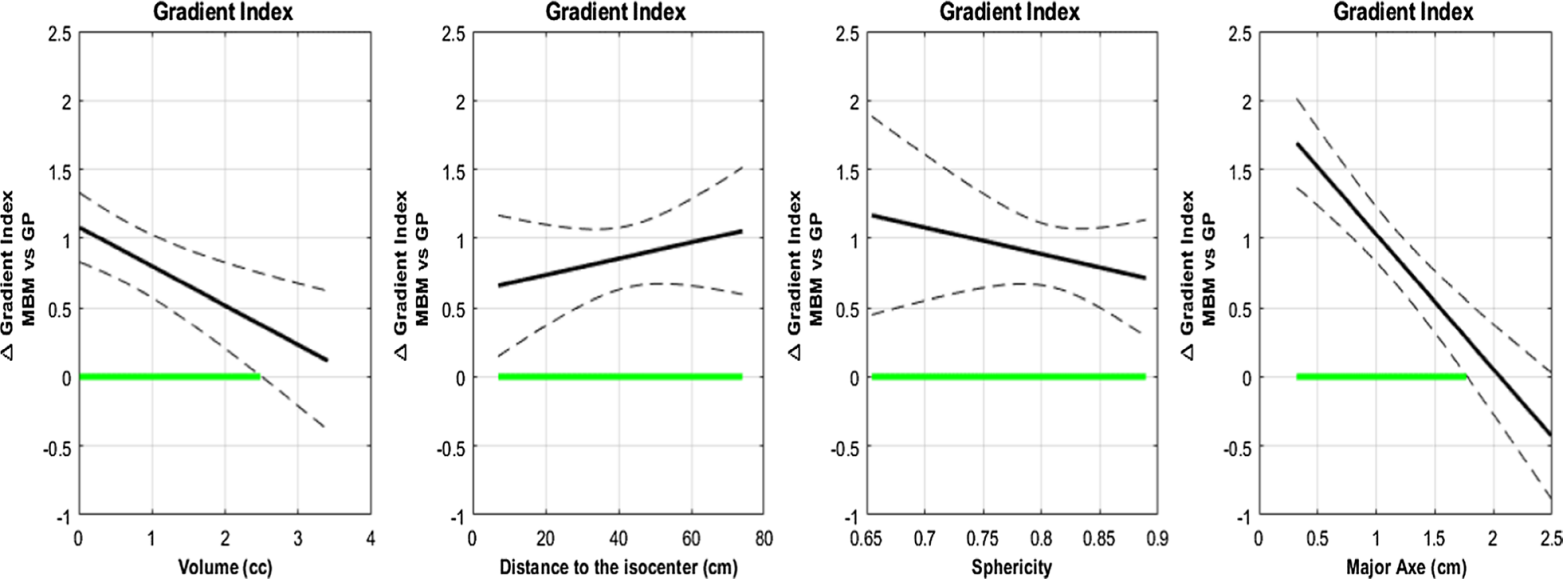
Gradient Index absolute difference between MBM and GP as a function of the volume, the distance to the isocenter, the Sphericity Index and the major axis diameter determined with a multivariate regression approach

The functional data analyses also highlighted this trend (Fig. 5). The difference between the mean MBM GI and mean GP GI decreased from 1 to 0 with the target volume and was not statistically significant for volumes higher than 2.5 cc. Considering the major axis diameter this difference decreased from 1.8 to − 0.5 and intersected the x-axis for a major axis diameter equal to 2 cm.

The mean MBM GI was significantly higher than the GP GI, irrespective of the sphericity index and the distance to isocenter considered (Fig. 5). With the sphericity index, the difference decreased from 1.1 to 0.8 whereas it increased from 0.6 to 1 with the distance to the isocenter.

##### Healthy brain tissue receiving at least 12 Gy

**Fig. 6:**
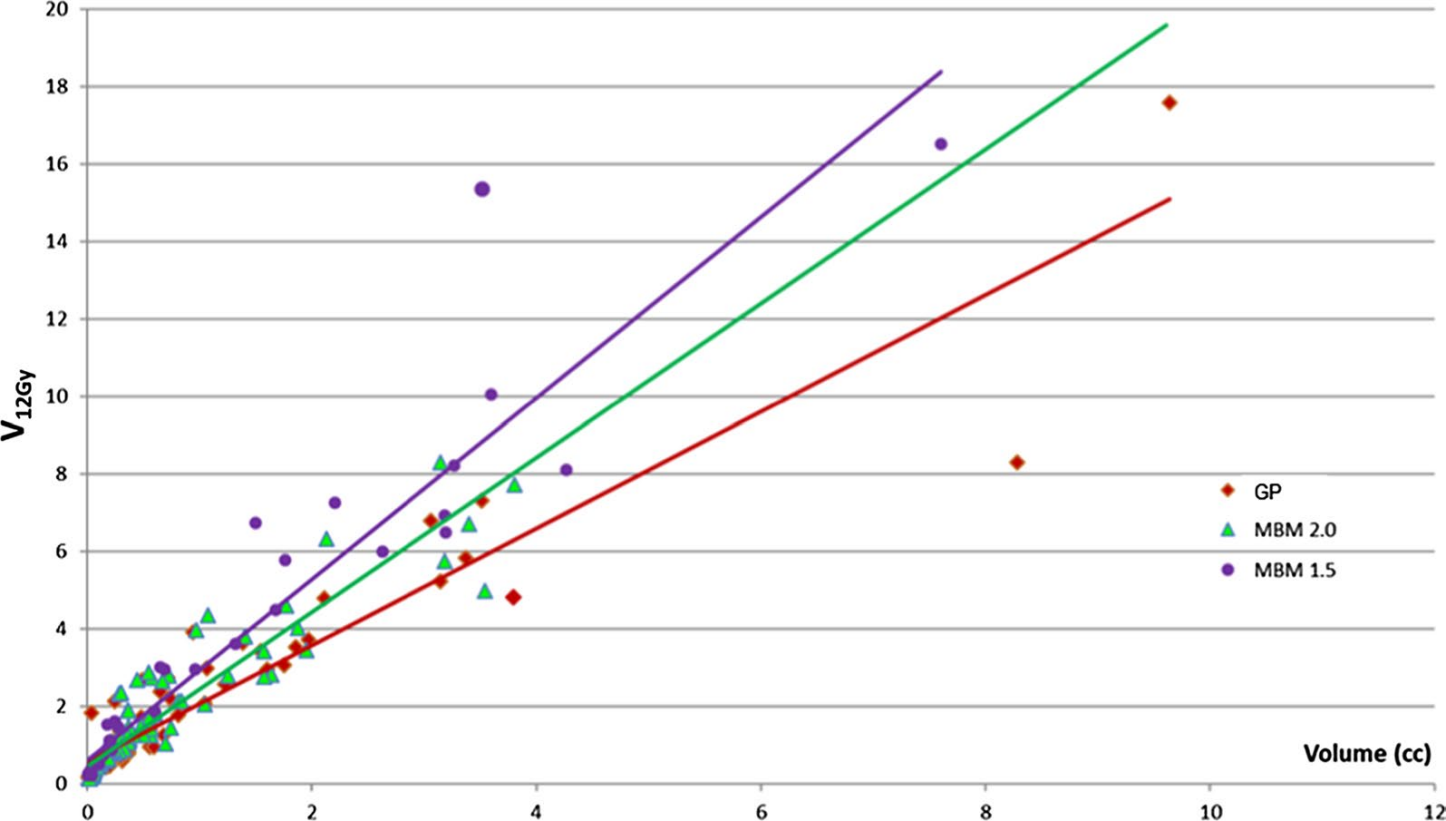
Normal Brain volume receiving at least 12 Gy considering the planning target volume for GP and MBM 2.0. MBM 1.5 data from our previous study [29] are reported

The overall mean V_12Gy_ was 1.87 ± 2.58 cc for MBM and 1.7 ± 2.32 for Gamma Knife (Table 4). The Fig. 6 represents the raw data of V_12Gy_ as a function of the target volume; the corresponding slope was 1.35 for Gamma Knife and 1.72 for MBM. This graph is clinically useful because it can determine approximatively the limitation of one kind of technique. The dose constraints regarding the healthy brain tissue were achieved for tumor volume below 5 cc with MBM version 2.0, and 6.5 cc for GP.

**Fig. 7:**
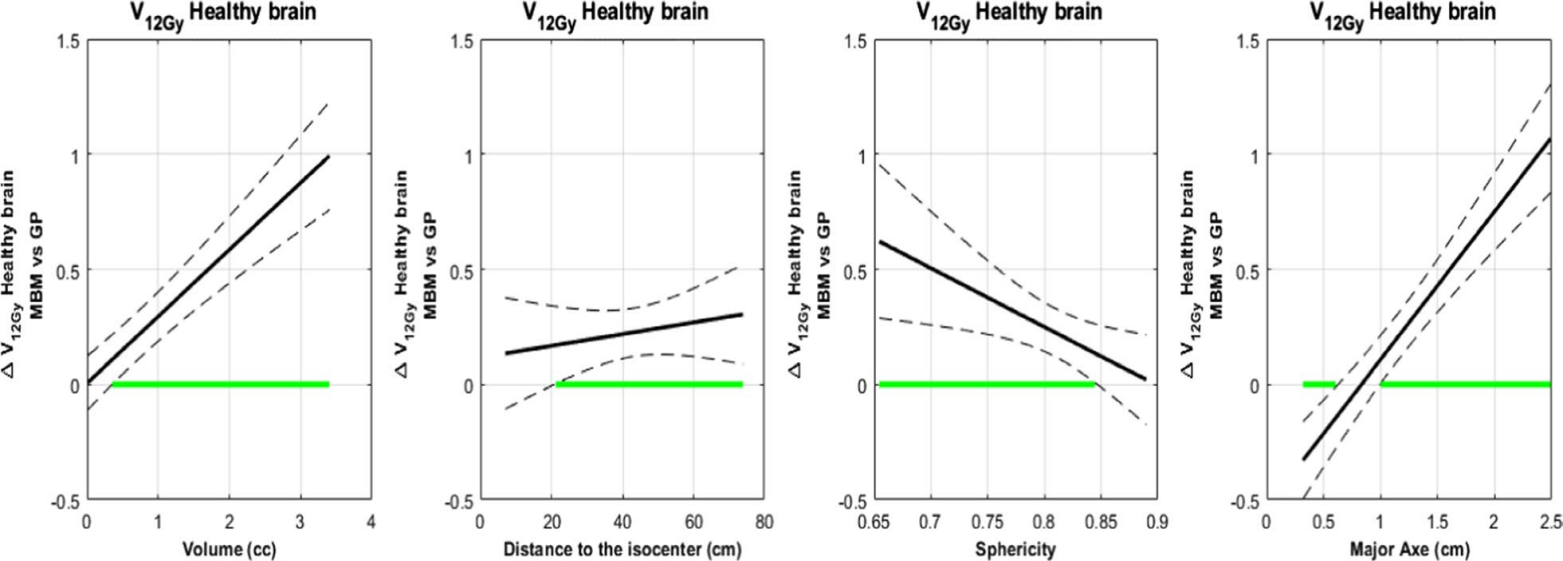
V_12Gy_ absolute difference between MBM and GP according to the volume, the distance to the isocenter, the Sphericity Index and the major axis diameter determined with a multivariate regression approach

For the volume dependency, the difference between the mean MBM V_12Gy_ and mean Gamma Knife V_12Gy_ increased from 0 to 1 cc and was statistically significant for volume higher than 0.4 cc (Fig. 7).

With the major axis diameter, this difference increased from − 0.4 to 1 cc and intersected the x-axis for a major axis diameter equal to 0.8 cm (Fig. 7).

With the sphericity index, the difference decreased from 0.6 to 0 cc and was significant below a sphericity Index of 0.84 (Fig. 7).

According to the distance to the isocenter, the difference between the mean MBM V_12Gy_ and the mean GP V_12Gy_ increased from 0.15 to 0.25 cc and was significantly higher for lesions located beyond 20 cm of the isocenter.

##### Organs at risk

Each of the plans met the OAR and normal tissue dose constraints. The MBM OAR doses were superior to the Gamma Knife doses but the differences were not found to be clinically relevant. Considering the very low doses and the minor differences observed, the results of the eyes, optic nerves and chiasma were not presented.

**Fig. 8:**
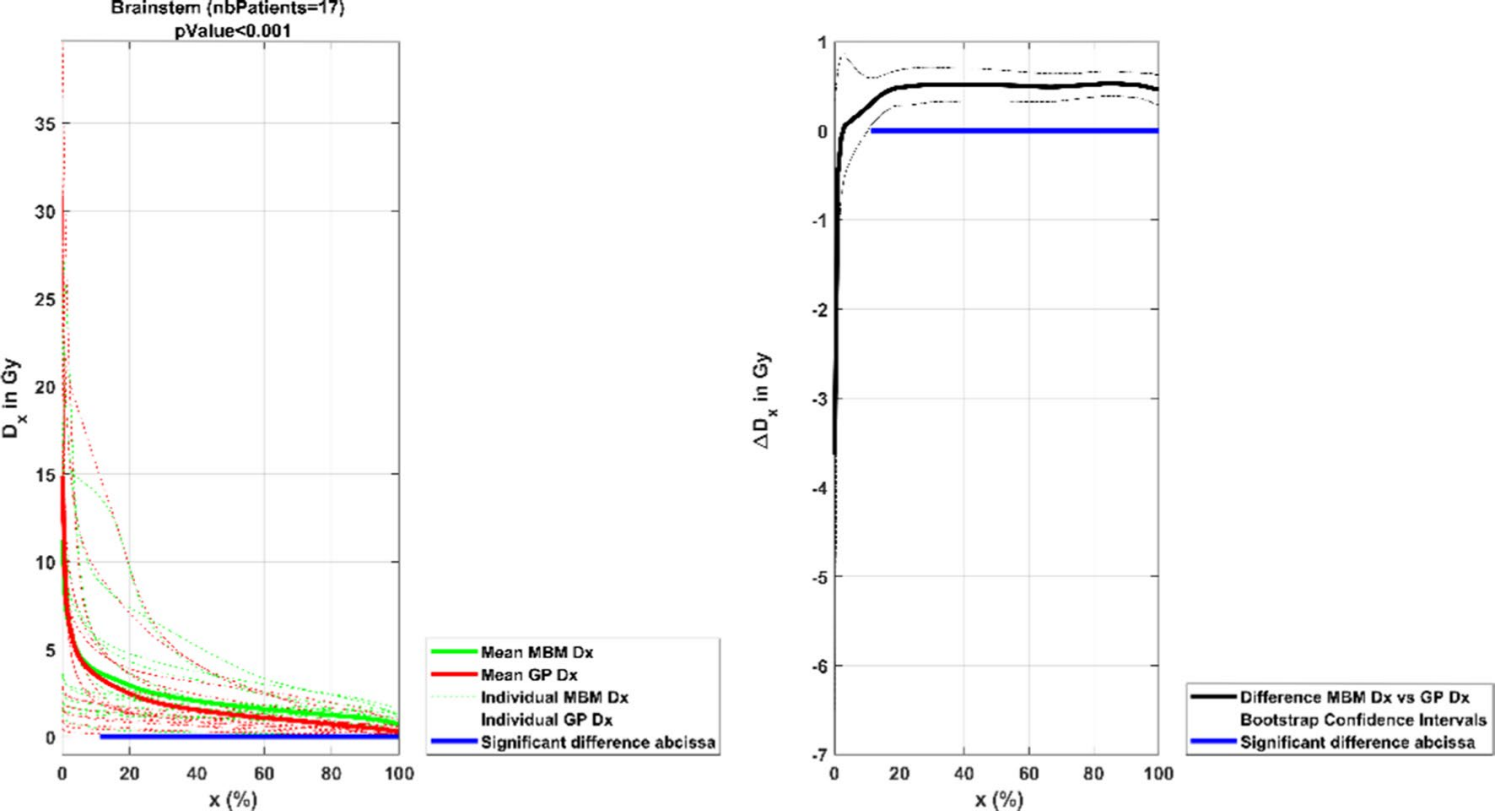
Mean DVH comparison between the MBM and GP brainstem plotted in dose as a function of the volume. The graph on the left illustrates each individual DVH (dotted lines), the mean GP DVH (red continuous line) and mean MBM DVH (green continuous line). The graph on the right shows the mean absolute dose difference as a function of the volume. The blue line represents the range where the difference is statistically different

For the brainstem (Fig. 8), for more than 10% of the organ volume, the difference between the mean MBM and GP doses was more than 0.5 Gy. The analysis below 10% of the volume included doses superior to 12 Gy, which corresponded to the aim to be reached in terms of maximum dose, the deviations were not statistically significant.

#### Targets close to the brainstem

##### Dosimetric indexes

**Fig. 9:**
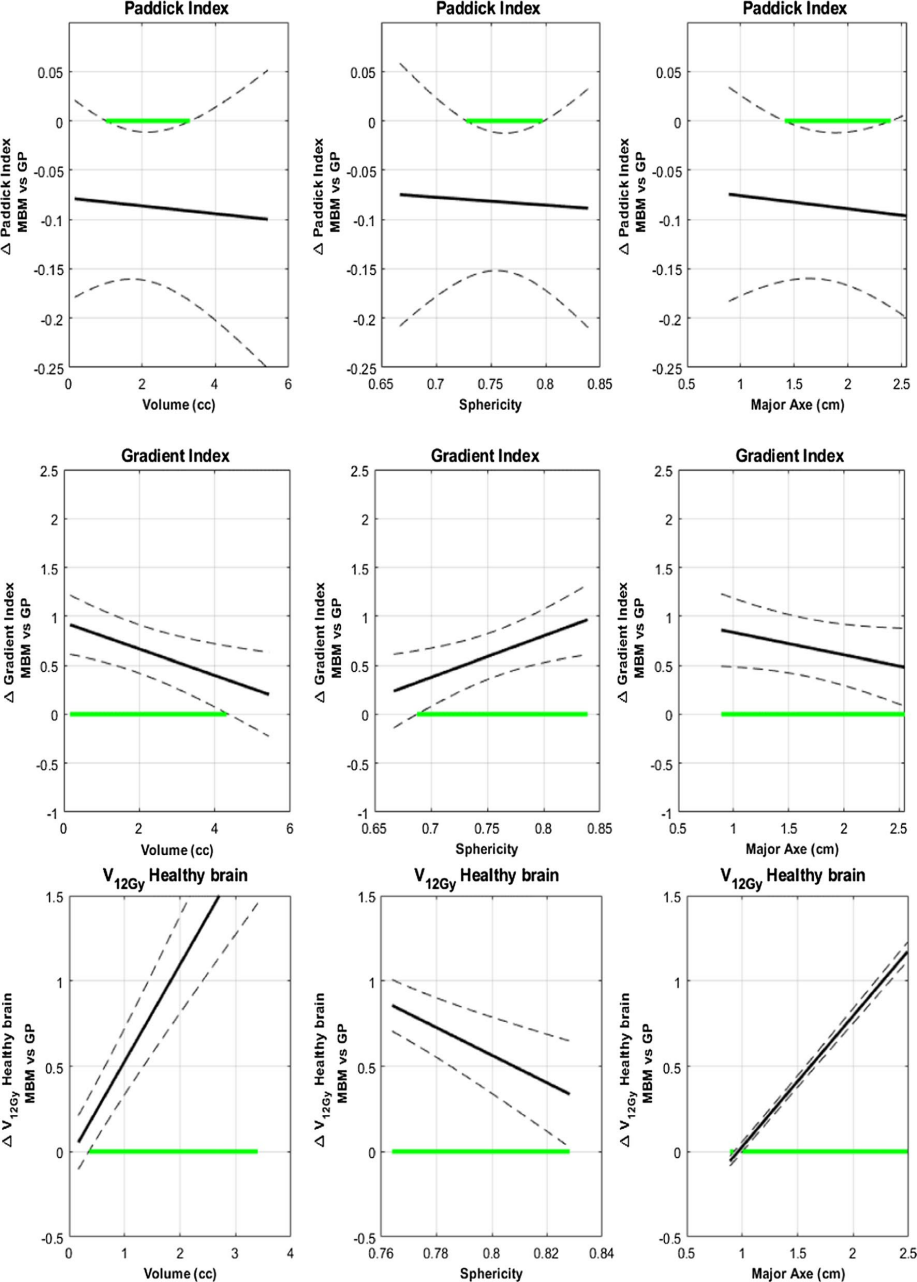
Paddick Index, Gradient Index and V_12Gy_ absolute difference between MBM and GP as a function of the volume, the distance to the isocenter, the Sphericity Index and the major axis diameter for the ten metastases close to the brainstem

By contrast with the overall study, the difference of the mean PI focused on the ten metastases close to the brainstem was always negative at − 0.1 with significance from 1 to 3.5 cc, a sphericity from 0.73 to 0.8 and a major axis diameter from 1.4 to 2.4 which means that the MBM PI was always worse in these specific cases. On the other hand, considering the whole cohort, MBM PI was improved for small metastasis with high sphericity in the overall study (Fig. 9). For complex cases, GP can perform plans with high selectivity but the time of planning and the treatment time were not considered in this focused study. The evolution of the GI and the V_12Gy_ were the same in both studies (Fig. 9).

For the ten selected patients, the mean volume of brainstem exposed at a dose higher than 12 Gy was 0.34 ± 0.38 cc for MBM and 0.19 ± 0.24 cc for GP (Wilcoxon signed rank test p = 0.02).

##### Dose fall-off

The Table 5 shows the dose fall-off expressed in millimeter. These raw data are interesting for knowing the distance necessary to decrease the dose from 20 Gy to an isodose line corresponding to an OAR constraint dose. The mean R_100-60_ refers to the distance from the isodose 20 Gy to 12 Gy useful for the brainstem toxicity; the mean distance was 2.2 ± 1.1 mm for GP and 2.6 ± 0.9 mm for MBM. The dose fall off was steeper in Gamma Knife plans, probably due to the larger number of beams.

**Table 5:** Mean dose fall-off distance to decrease from the isodose prescription 100 to 80, 60, 50 and 20% towards the OAR direction or not for the targets close to the brainstem

	Mean R 100–80 (mm)		Mean R 100–60 (mm)		Mean R 100–50 (mm)		Mean R 100–20 (mm)	
	GP	MBM	GP	MBM	GP	MBM	GP	MBM
No OAR	0.9 ± 0.5	1.2 ± 0.3	2.1 ± 1.1	2.6 ± 0.9	2,9 ± 1.6	3.6 ± 1.4	8.3 ± 5.5	12,3 ± 7
OAR	0.9 ± 0.4	1.1 ± 0.2	2.3 ± 1.2	2.5 ± 0.6	3.3 ± 1.7	3.5 ± 1.2	9.7 ± 5.3	11.4 ± 5.4
Total	0.9 ± 0.5	1.2 ± 0.3	2.2 ± 1.1	2.6 ± 0.8	3 ± 1.6	3.6 ± 1.3	8.6 ± 5.4	12 ± 6.6

The dose fall-off expressed in %/mm is the most appropriate definition and can give an order of magnitude of value comparable to other techniques already published.

**Fig. 10:**
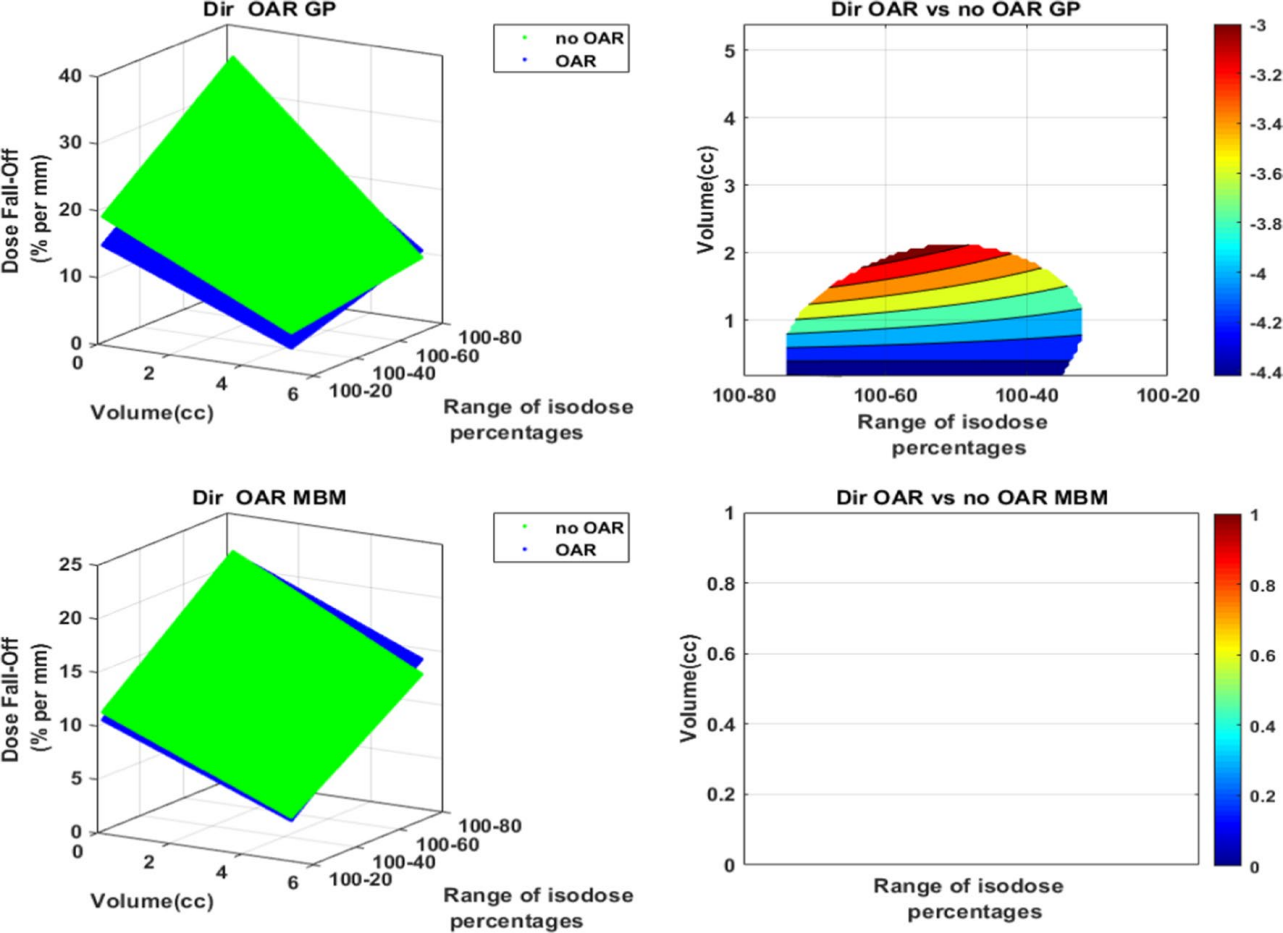
Dose fall-off (%/mm) representation as a function of the volume and the isodose line in the OAR direction versus the “No OAR” direction. The figures on the left illustrate in 3D the dose fall-off absolute values as a function of the volume of the PTV and the isodose percentages selected. The graphs on the right indicate the absolute dose fall-off difference. The white areas are for differences not statistically significant

The presence of an OAR close to the PTV had no impact on the dose fall-off: no improvement of the dose fall off value was observed neither with MBM nor GP. The dose fall off was even inferior on GP for lesions smaller than 2 cc (Fig. 10).

**Fig. 11:**
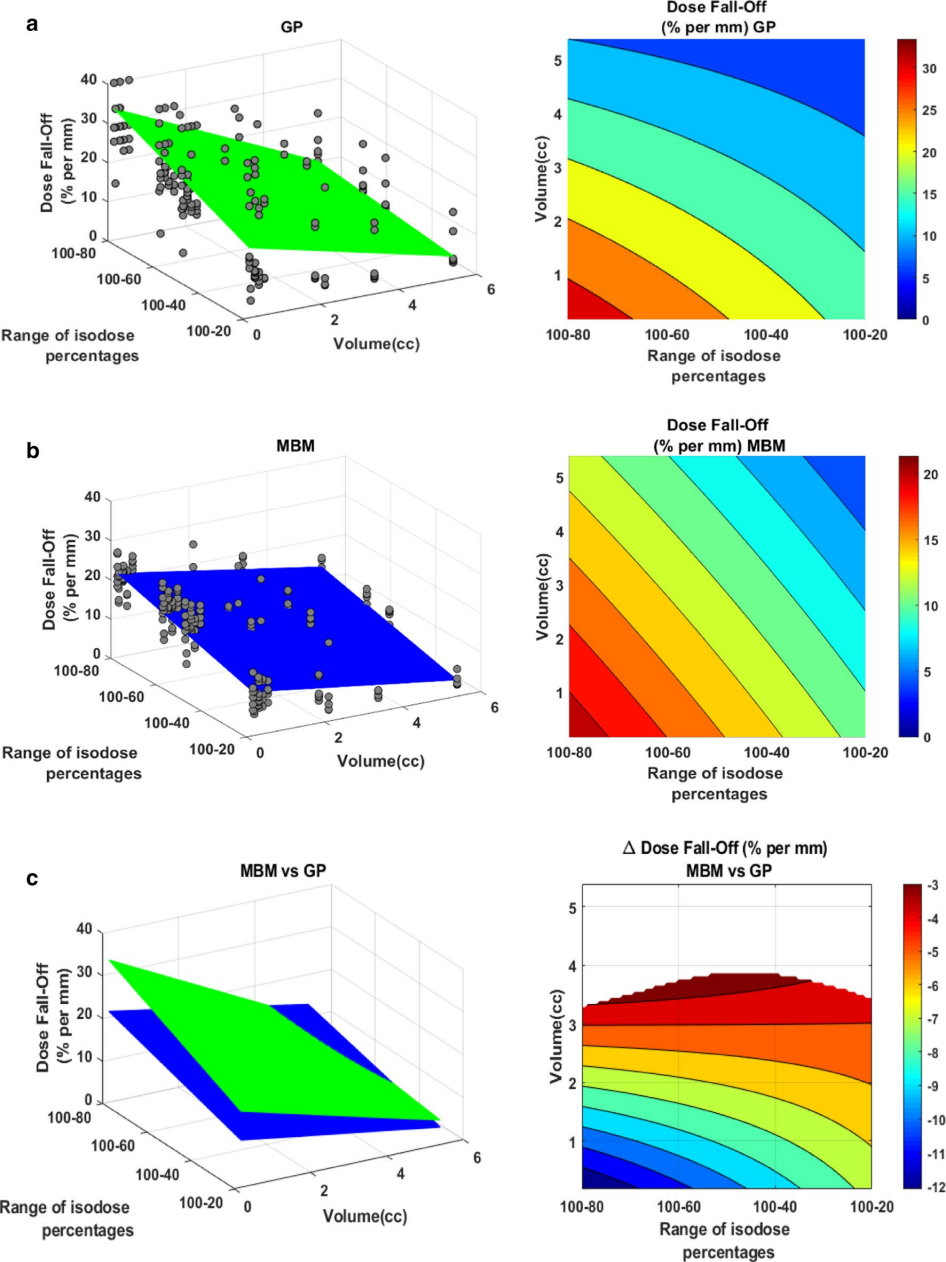
Dose fall-off (%/mm) representation as a function of the volume and the isodose line for GP (**a**) and MBM (**b**). Figure (**c**) illustrates the GP vs MBM dose fall-off: the left figure illustrate in 3D the dose fall-off absolute values as a function of the volume of the PTV and the isodose percentages selected, the right graph indicates the absolute dose fall-off difference. The white areas are for differences not statistically significant

For both techniques, the mean dose fall off decreased with the volume and the isodose value (Fig. 11). For GP, it ranged from 5 to 30%/mm whereas it ranged from 4 to 20%/mm for MBM. The MBM dose fall-off was significantly lower for lesions smaller than 3.8 cc, this deviation increased when the volume and the isodose decreased to reach a maximum of − 12%/mm.

**Fig. 12:**
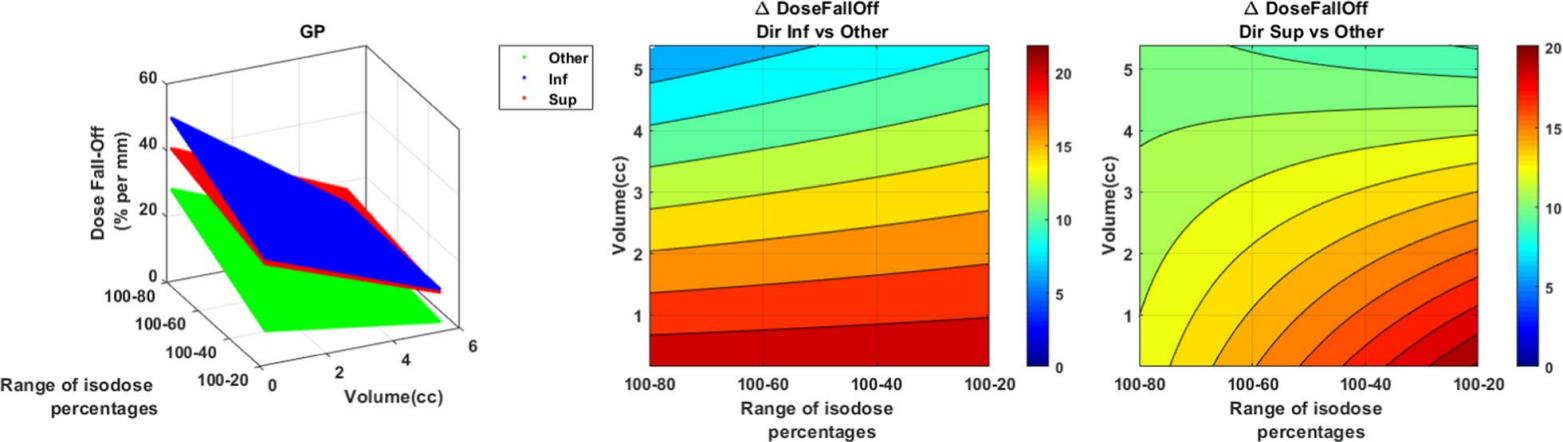
GP dose fall-off (%/mm) representation as a function of the volume and the isodose line in the superior and inferior direction versus the “others” direction (anterior, posterior, left and right). The figure on the left illustrate in 3D the dose fall-off absolute values as a function of the volume of the PTV and the isodose percentages selected. The right graphs indicate the absolute dose fall-off difference

The GP dose fall-off had a preferential orientation (Fig. 12). In the inferior direction, the difference with the 4 other directions (anterior, posterior, right and left) decreased with the volume and was higher from 5 to 20%/mm. In the superior direction, the deviation with the 4 other directions was better of 6 to 20%/mm. The difference increased with the isodose and when the volume decreased.

**Fig. 13:**
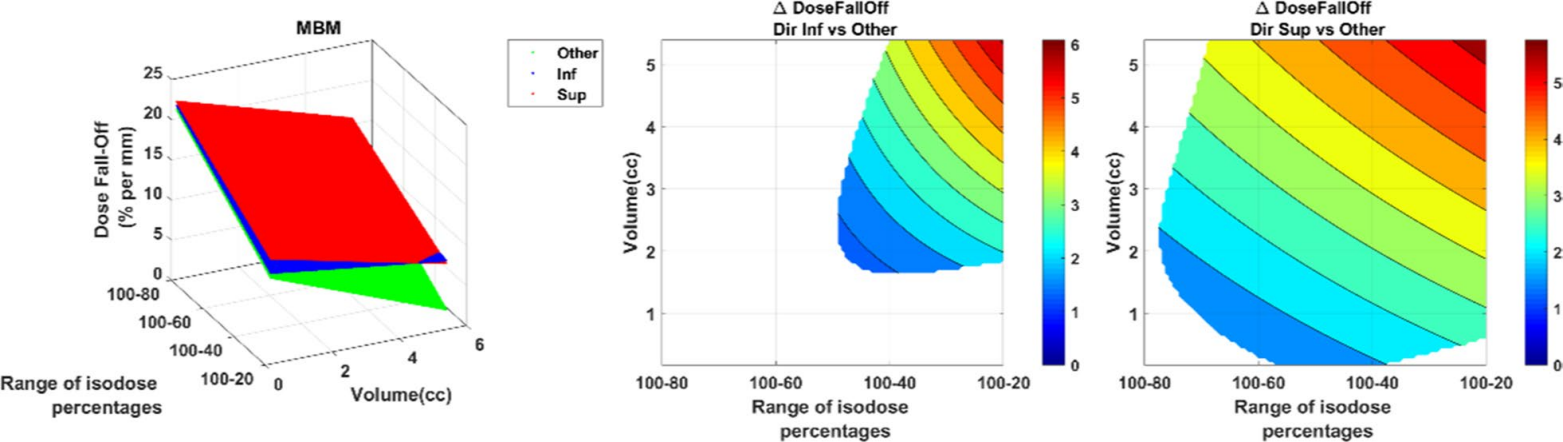
MBM dose fall-off (%/mm) representation as a function of the volume and the isodose line in the superior and inferior direction versus the “others” direction (anterior, posterior, left and right). The figure on the left illustrate in 3D the dose fall-off absolute values as a function of the volume of the PTV and the isodose percentages selected. The right graphs indicate the absolute dose fall-off difference. The white areas are for differences not statistically significant

For MBM, the effect of the direction was less evident (Fig. 13). For the inferior direction, the difference was significantly higher only for volume larger than 2 cc and isodose 50% with a maximum difference at 5%/mm. For the superior direction, the difference with the 4 other directions increased with the volume and the isodose to reach a maximum at 6%/mm.

##### Treatment time

All the GP plans were recalculated on 04/11/2019, the mean beam on time was 169 ± 47.5 min and rescaled to the date of the cobalt source reloading so the GK beam on time was divided by 1.8 and equal to 94 ± 26.4 min (Wilcoxon signed rank test *p* < 0.001). MBM beam on time was 13 ± 3.2 min so the beam-on time is divided by 7.2 compared to the GP plans (Wilcoxon signed rank test *p* < 0.001).

## Discussions

Despite two different approach of beam delivery (beam energy, number of isocenters) and treatment planning (manual or automatic), both techniques can achieve high quality treatment plans required for SRS. The impact of the target volume geometrical characteristics could have been assessed in isolation highlighting the importance of the volume effect.

Unlike GP, the use of MBM will obviously require margins applied to GTV to generate PTV mainly due to the delivery uncertainties of the linac and the positioning rotational residual errors. This consideration will probably affect the V_12Gy_ and the OAR doses but to compare strictly the two techniques, the present study was performed without margin. The margin used on a linac for a mono-isocentric technique can be of 0, 1, 2 mm or even non-uniform [23] depending on the distance from the isocenter and the repositionning tolerance. Considering the possibilities in GP, it's also possible to add margins so we cannot exclude that some groups use a margin on the GP.

Using a monoisocentric technique, any rotational residual errors will be amplified with subsequent off-axis localization errors and can be a concern to take into account with consequences in clinical practice. Over 7–8 cm from isocenter, there is a need to add 1 mm margin [24, 25]. Prentou et al. [26] evaluated the dosimetric impact of simulated rotational positional errors for multiple metastases VMAT SRS cranial cases. For single isocenter plans and 1° rotation, the plan quality indices were significantly deteriorated for targets distant of more than 4 cm from the isocenter. For 2° rotation, the conformity index was deteriorated by on average 7.2%/cm and 2.6%/cm when two isocenters are used. Faught et al. [27] demonstrated that the linac mechanical uncertainties have also an impact on off axis targets with monoisocentric treatments. They studied the influence of induced errors on collimator, couch and gantry rotations at TG 142 tolerance levels. For multimet single isocenter VMAT plans, 1° of collimator rotation led to an average change to V100% and D99% of 5% and 6% respectively. In the present study, only one case has required two isocenters because of two targets in extreme positions with a distance of 15 cm from each other. The treatment planning performed with one isocenter presented a poor selectivity. All these recommendations depend on the 4D or 6D correction, the repositioning tolerances and the mechanical tolerances and uncertainties of the linac.

Some publications showed the benefit of using a 6 MV FFF photon beam instead of a 6MV flattened beam in case of stereotactic treatments especially in terms of treatment time.

Viellevigne et al. [28] compared the use of a 6 MV and a 6 MV FFF for SBRT with DCA. They found a slight improvement of the conformity and the healthy tissue protection for a 6 MV FFF beam especially for small volumes. With FFF beams, they had difficulties to cover and maintain a good homogeneity for large volumes because of the beam inhomogeneity and the MU were significantly increased. However the treatment time was considerably reduced due to the high dose rate (1400 MU/min vs 600 MU/min). Dzierma et al. [29] compared 7 MV FFF to 6 MV plans with quality indices, dose to OAR and out-of-field dose, in case of intracranial radiosurgery for multiple metastases with multiple non coplanar arcs. The 7 MV FFF plans were marginally superior to the flat beam 6 MV plans with treatment times reduced almost by half. These studies were led with targets on the isocenter but due to the profile inhomogeneity, these conclusions can’t be applied on a monoisocentric unmodulated technique. We didn’t find any study showing the benefit of a 6 MV FFF beam on the plan quality for such a technique. The 6 MV FFF beam was not available in our TPS to investigate this point in our study. The only clear advantage is the considerably reduced beam-on time, an important parameter for the intra-fraction motion management.

To our knowledge, two articles can be found currently for MBM version 2.0; one was published by Kuntz et al. They compared MBM version 2.0 to VMAT plans and they achieved very high conformity values with PI around 0.85 whereas our mean PI is 0.57 ± 0.12. The volume of the lesions considered in both studies can explain this difference. Kuntz et al. had mean GTV + 1 mm = 2.3 cc against 0.76 cc in our study. In the present study, we observed that the volume of the target strongly influences the PI. Moreover, the PTV coverage objectives were not the same: 95% of the PTV must be covered by D_98%_ whereas we obtain 100% covered by D_100%_ in our cases. The PTV volume differences could also explain the differences in terms of mean GI: 3.55 ± 0.59 in the article published by Kuntz et al. compared with 4.09 ± 1.14 reported in our work. The other dosimetric study available with MBM version 2.0 performed with HD120 MLC by Taylor et al. also achieved a better PI and GI than our study. The target volumes were also higher than our study (2.16 cc vs 0.76 cc) and could explain those differences.

More published data are available for MBM version 1.5. The mean absolute values of PI and GI are improved compared with our study’s results but the mean volume of the lesions are still higher with different PTV coverage purposes [2, 5, 7, 8]. Narayanasami et al. [30] is the only study with PTV mean value close to those observed in our series: 0.7 vs 0.76 cc in our work. They found a PI 0.555 using MBM v1.5 which is comparable to our value of 0.57. Concerning the GK PI values obtained in our investigation, owing to the fact that we plan without margin, our clinical practice is to ensure the coverage to compensate the localization uncertainties.

The only dosimetric comparison between the Gammaknife and the MBM version 1.5 with enough statistics is the study led by Vergalasova et al. [5] MBM 1.5 had poorer PI and GI especially for sizes smaller than 1 cm. We still obtain a worse GI for small lesions but PI is better for small targets in our study for the version 2.0.

Hofmaier et al. shows statistically significant correlations sphericity and V_12Gy_ for MBM version 1.5 compared with VMAT plans and performs good healthy brain tissue sparing for high sphericity. In the present study, high sphericity is also favorable to MBM version 2.0 with a higher PI and a comparable V_12Gy_ to GP.

Our previous work based on version 1.5 [31] showed equivalent selectivity but revealed difficulties to spare healthy tissues and OAR making the single isocenter dynamic conformal arc therapy (SIDCA) an interesting technique in terms of treatment time for patients with small lesions distant from each other. The latest version of MBM provides optimization algorithms to considerably reduce the healthy brain volume receiving more than 12 Gy and to cut V_12Gy_ bridges between two brain metastases close to each other. A Normal Tissue Sparing (NT) slider in the graphical user interface can be used to control the dose gradient optimization. Increasing normal tissue sparing will typically decrease the beam´s eye view margins around each target, increasing the heterogeneity inside the target and reducing the dose gradient outside the target. The V_12Gy_ exceeded 10 cc for PTV bigger than 2.5 cc in the MBM 1.5 version compared with 5 cc using the MBM 2.0 version. In the present study we were able to select patients with multiple lesions close to each other and to the brainstem. The study led by Taylor et al. also confirmed the plan quality improvements of the version 2.0 compared to the version 1.5 in terms of selectivity and dose spillage.

The arrangement of the HD MLC seems to have a low influence on the plan quality. In our study, the PI is constant despite larger leaves after 4 cm and the slightly GI increase slighly with the distance to isocenter. Stanhope et al. [32] showed a degradation of the quality plan (Conformity index and gradient index) for VMAT plans especially for small lesions with volume lower than 1 cc. Taylor et al. [9] also found an influence of the MLC design with a significant improvement of the plan quality. For the version 2.0 of MBM, the mean conformity index increased from 0.79 to 0.82, the mean gradient index improved from 3.76 to 3.15 and the mean volume normal brain receiving more than 12 Gy decreased from 16.15 to 13.72 cc when they switched from the Agility MLC of Elekta with leaves of 5 mm wide to the Varian 120 HDMLC with central leaves of 2.5 mm wide.

Figure 6, shows the volume of healthy brain receiving at least 12 Gy depending on the target volume but these results are a little bit overestimated because they are noted regardless of the number of lesions. Saghal et al. [33], found a significant increase in 12 Gy volume for multiple target treatments compared with single target treatments by approximately 4% per target when a high dose such as 20–24 Gy was used.

In our investigation, the PI only take into account PTV percentage covered by the prescription isodose. Dimitriadis et al. proposed the Efficiency Index to assess the treatment plan quality in SRS [34]. It combined conformity, gradient and mean dose into a single value and was adaptable to multiple target plans. This index is favorable to the GK because it takes into account the PTV mean dose and the V_10Gy_ for the numerator and the denominator respectively.

Munshi et al. described a sharpest dose fall-off in the cranio-caudal direction in accordance with our results which indicates that this effect strongly depends on the treatment delivery because the 3D radiotherapy and the VMAT cases were performed with restricted couch angles. The preferential orientations can also be explained by the Gammaknife beam delivery method which is made up with 192 cobalt sources arranged in crown. The higher dose fall off found in regard of OAR is produced by the beam arrangement of the 3DRT and the ability of the VMAT to spare OAR. On the other hand, the techniques studied in our work seem to spare OAR and normal tissues by reducing the overall dose spillage with high heterogeneity inside the PTV and not by blocking one beam direction. The mean 3DRT/VMAT dose fall off 100–80, 100–50 and 100–20 were 7.0 ± 1.5, 4.7 ± 0.8 and 4.1 ± 1.6%/mm respectively. These results are much lower compared to our data. The GK dose fall off 100–80, 100–50 and 100–20 was 27.2 ± 12.1, 21.6 ± 11.3 and 14.4 ± 10%/mm and the MBM dose fall off 100–80, 100–50 and 100–20 18.1 ± 4.3, 15.5 ± 4.7 and 8.5 ± 3.9%/mm. This can be explained by the considered PTV volume, Munshi et al. mean PTV volume was 11.7 ± 16.1 cc whereas our mean PTV volume was 1.47 ± 1.75 cc. The Fig. 11 illustrates the strong decrease of the dose-fall off with the tumor volume.

In our study, none of the techniques shows a strong ability to protect OAR. For GP plans, there is a possibility to have strong gradient in one direction by plugging some shots but the treatment time is considerably increased and the isodose resulting, distorted. In our Institution, we plug the shots to protect small structures with high constraints like cochlea in acoustic schwannoma treatments. For our ten cases selected, the brainstem surrounds a large part of the metastasis making the plugging not really relevant.

The gradient index is commonly used to characterize the dose spillage but our study reveals the limit of this index as a good indicator of the quality plan for the dose fall-off and the V_12Gy_ absolute value. In the dose fall-off analysis, compared to the Fig. 4, the results are conflicting because they both decrease with the volume. Moreover, the mean V_12Gy_ deviation between GP and MBM increase with the volume (Fig. 6) whereas GI tends to become comparable with increasing volumes. This can be explained by the predominance of the PIV in the denominator part and his dependence to the selectivity [35]. In the parallel study, we expected that the Gradient Index and the V_12Gy_ according to the IDL prescription would follow the same trend but the results are not correlated. This difference is counter-intuitive. Indeed the V_12Gy_ is a global data which ponders the gradient by the selectivity. For small lesions treated with MBM, the selectivity is improved, the gradient is worse and V_12Gy_ lower than using GP. For large metastases treated on GP, the selectivity is better, the gradient is equivalent and the V_12Gy_ is lower than MBM. For high sphericity, with MBM, selectivity is higher, the gradient is worse and the V_12Gy_ equivalent to the GP plans. So in this series, the V_12Gy_ seems to rather be correlated to the selectivity. In summary, for the comparison, the Gradient Index does not reflect the dose spillage for two targets or two groups of targets of different volume. The comparison is relevant only if the lesions are equivalent in terms of volumes and selectivity.

## Conclusions

For the mono-isocentric linac-based radiosurgery, the isodose line prescription around 70–75% was the best compromise between selectivity and sparing tissues. For both techniques, the selectivity and V_12Gy_ increased with the target volumes while the gradient and dose fall-off decreased. In our series, the dose fall-off and the V_12Gy_ were more relevant indicators than the Gradient Index for the low dose spillage assessment. Both evaluated techniques can achieve a high level of selectivity and dose fall-off, essential to the radiosurgery with preferred orientations according to the beam delivery technique. Overall, both produce comparable plan qualities with a slightly improved selectivity with MBM for smaller lesions but with healthy tissue and OARs sparing slightly favorable for GK at the expense of a considerably longer irradiation time. However, a higher healthy tissue exposure must be considered for large volumes in MBM plans.

## Data Availability

The datasets supporting the conclusions of this article are included within the article.

## Abbreviations

CI: Conformity Index
CT: Computed tomography
CTV: Clinical target volume
DVH: Dose volume histogram
FFF: Flattening filter free
GI: Gradient index
GP: Gamma Plan
GTV: Gross target volume
IDL: Isodose line
MBM: Multiple Brain Mets
MIDCA: Multiple isocenter dynamic conformal arcs
MLC: Multi leaf collimator
MU: Monitor unit
MRI: Magnetic resonance imaging
OAR: Organ at risk
PI: Paddick index
PTV: Planning target volume
SI: Sphericity index
SRS: Stereotactic radiosurgery
SRT: Stereotactic radiotherapy
VMAT: Volumetric modulated arc therapy
V_12Gy_: Healthy brain receiving at least 12 Gy
WBRT: Whole brain radiotherapy
